# High Temperature Measurement with Low Cost, VCSEL-Based, Interrogation System Using Femtosecond Bragg Gratings

**DOI:** 10.3390/s22249768

**Published:** 2022-12-13

**Authors:** Konrad Markowski, Juliusz Bojarczuk, Piotr Araszkiewicz, Jakub Ciftci, Adam Ignaciuk, Michał Gąska

**Affiliations:** 1 Institute of Telecommunications, Warsaw University of Technology, Nowowiejska 15/19, 00-665 Warsaw, Poland; 2 FiberTeam Photonic Solutions, Warszawska 102, 20-824 Lublin, Poland; 3 Faculty of Materials Science and Engineering, Warsaw University of Technology, 141 Woloska St., 02-507 Warsaw, Poland; 4 Faculty of Physics, Warsaw University of Technology, Koszykowa 75, 00-665 Warsaw, Poland

**Keywords:** VCSEL, HCG, interrogator, bragg grating, fbg, high temperature

## Abstract

In this article, a cost-effective and fast interrogating system for wide temperature measurement with Fiber Bragg Gratings is presented. The system consists of a Vertical Cavity Surface Emitting Laser (VCSEL) with a High Contrast Grating (HCG)-based cavity that allows for the fast tuning of the output wavelength. The work focuses on methods of bypassing the limitations of the used VCSEL laser, especially its relatively narrow tuning range. Moreover, an error analysis is provided by means of the VCSEL temperature instability and its influence on the system performance. A simple proof of concept of the measurement system is shown, where two femtosecond Bragg gratings were used to measure temperature in the range of 25 to 800 °C. In addition, an exemplary simulation of a system with sapphire Bragg gratings is provided, where we propose multiplexation in the wavelength and reflectance domains. The presented concept can be further used to measure a wide range of temperatures with scanning frequencies up to hundreds of kHz.

## 1. Introduction

The fast and dynamic growth of various industries is accelerating the development of sensing systems, especially in terms of their capacity, speed of operation, and accuracy. The still-evolving Industry 4.0 requires detailed and wide information about physical parameters such as strain, gas concentration, temperature, and others to implement autonomous control and improve remote diagnostics [1,2,3,4]. For example, precise information on the temperature in electrical energy transformers can lead to the early detection of a fault or overload [5,6], which can help to plan maintenance intervals. Moreover, temperature and strain measurements can help detect battery swelling or be used for the optimization of the power packs’ structure, which is beneficial for the development of electromobility [7,8,9,10]. In most applications that operate within a harsh and challenging environment, researchers and companies around the world find that fiber Bragg gratings (FBGs) are an excellent choice for multiparameter sensing [11,12].

The technology of optical fiber sensors has been developing for decades [13,14,15]. Due to the fact that the Bragg grating sensor is inscribed in the optical fiber and operates on light, it is naturally immune to the harsh environment and severe conditions. This is why these sensors have found many applications in a wide list of industries. For instance, FBG was found to be great for both temperature and strain measurement [13], monitoring of power infrastructure [16], military applications or even monitoring components for space missions [17,18]. Significant interest is put into high-temperature sensing, especially in harsh applications, where fiber-based sensing is one of a few reasonable choices. Indeed, most of the presented research applies FBGs to measure temperatures up to hundreds of Celsius degrees. However, there are still some applications that require much wider temperature ranges and thus optimization of the whole measurement systems [19,20]. Since Hill reported the photosensitivity effect in doped fibers, many types of FBG structures have emerged, especially femtosecond gratings [21,22] and functionalized gratings that can be produced by coating the optical fiber with different chemical compounds or polymers [23,24,25]. The current development of FBG technology is mostly focused on sensing applications [26] and great interest is shown in the field of comprehensive sensing systems based on optical technology [27,28,29].

However, it is still observable that current optical-based sensing systems cannot compete with standard electronic solutions mostly because of their price. FBG-based sensors are naturally passive elements, so they must be stimulated by an incident light wave, which is conducted by the interrogator, and it contributes to the major part of the cost of the sensing system. Previously reported systems are based on the use of a broadband light source (BBS) and photodetector (PD) array [30]; BBS and tuned filters [31,32,33,34]; tuned laser source and PD [35,36,37]; and others [38,39,40,41]. All of the approaches presented cannot be widely used in the industry due to the utilization of expensive optoelectronic elements. For Industry 4.0 applications, the most beneficial would be either the usage of temperature/current-swept lasers or photonic-integrated circuits [42,43]. The first solution, when provided in a small, cost-effective package, typically offers a relatively small spectral range, which renders it not applicable in terms of a wide measurement range. What is more, temperature sweeping offers relatively slow interrogating times, while current-based sweeping can result in the highly unwanted mode hopping of the light source. The second solution is still emerging and requires more research in order to create cost-effective interrogating devices.

In this article, an interrogation system based on Vertical Cavity Surface Emitting Laser (VCSEL) with High Contrast Grating (HCG) is presented. The VCSEL is used to interrogate FBGs in the range of up to 10 nm without mode hopping and offers a sweeping speed of up to a hundreds of kHz [44]. The main focus of this work is put on an analysis of a system for wide temperature measurement that bypasses the limited tuning range of the HCG-VCSEL. The presented approach benefits from its simplicity, and low error, while its temperature range measurement is restricted only to the limits of the FBG capabilities. By the proper adjustment of the initial Bragg wavelength of each grating, it is possible to overcome the tuning limits of a VCSEL laser. For the most demanding applications, a system with multiplexation in both the wavelength and reflectance domains is presented, where high-temperature FBGs can be used. Since in the experiment a laser without temperature stabilization is used, an error of a central wavelength of the sensor analysis in the basics of laser temperature deviation is performed. The presented concept can be further used in many applications including, but not limited to high temperature measurement, analysis of battery cells, or medicine [45,46].

## 2. Principle of Working

**Figure 1:**
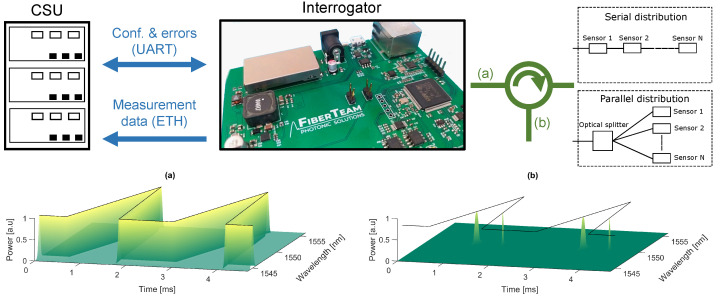
Architecture of the measurement system. Communication between CSU and the interrogator is based on Ethernet and USB-UART. The interrogator can be used to analyze serially or parallelly distributed sensor networks. In (**a**), the simulation of an output signal in wavelength and time domains is shown. Meanwhile, in (**b**), the simulation result of a received signal from one FBG sensor is presented.

The architecture of the measurement system is presented in Figure 1 and consists of the Control & Storage Unit (CSU), the interrogator, and the FBG-based sensor network. The CSU is connected to the interrogator via Ethernet and USB-UART and is responsible for both storing and controlling the measurements made by the interrogator. The sensor network consists of *N* FBGs, which are suitable for the desired application, e.g., high-temperature measurement. The main component of the whole system is the optical interrogator, which is responsible for active spectrum analysis.

### 2.1. FBG Sensor Network

In order to properly analyze the system performance, let us describe the optical fiber sensor network by means of the principle of operation. One can distinguish two general types of FBG sensing networks. The first and most common is the configuration of serially distributed Bragg gratings.

The second type of sensor network consists of parallelly distributed FBGs, where the received power spectrum of the sensors can be simplified to the sum of the power reflected from each sensor. Nevertheless, the selection of an FBG distribution should depend on the user’s needs along with the desired sensing capabilities. In further deliberations, it is assumed that the FBG sensor network consists of serially distributed optical FBGs inscribed on a single fiber.

**Figure 2:**
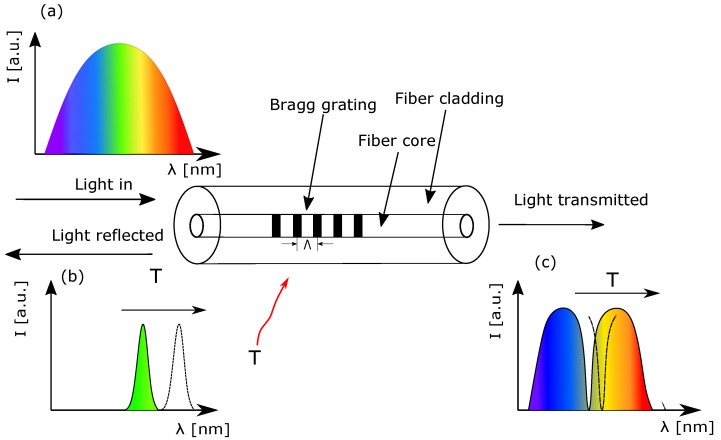
Operating principle of the fiber Bragg grating. The FBG works as a wavelength-domain filter that reflects the narrowband of an incident spectrum. When external stressors act on FBG, its reflection spectrum changes. In subfigure (**a**), an incident spectrum is shown. In (**b**,**c**), a reflected and transmitted spectrum are presented, respectively.

The Fiber Bragg Grating is a periodic structure that arises from local changes in the refractive index of a fiber, whose source defines the type of such structure [47]. When the light wave interacts with periodic changes in the refractive index of a fiber, a self-coupling of light occurs. Undoubtedly, this coupling will strongly depend on the period of changes in the refractive index and the wavelength of the incident wave [48]. The Bragg wavelength for which the resonance between two counter-propagating modes occurs can be calculated from Equation (1).

(1) $$\lambda_{B}=2n_{eff}\Lambda ,$$

where $n_{eff}$ denotes the effective refractive index of an optical fiber and Λ is a period of index changes in the fiber. The principle of operation of the Bragg grating is presented in Figure 2.

It is worth noting that FBGs are justifiably used as different types of sensors. When, e.g., temperature acts on the optical fiber, two main effects are observable. The first, thermal expansion, affects the change of Λ of a grating as a function of temperature, while a thermo-optical effect causes the change of $n_{eff}$ of an optical fiber and is more influential than the thermal expansion of the glass [49]. The general formula that describes the change of a Bragg wavelength as a function of temperature is given by Equation (2).

(2) $$\Delta \lambda_{B}\left(T\right)=\alpha \left(T-T_{0}\right)+\beta {\left(T-T0\right)}^{2},$$

where α denotes the linear change, and β describes the quadratic change of a wavelength as a function of temperature. For a relatively low-temperature measurement range, β can be neglected, but for a much wider temperature analysis, it must be taken into account since optical fiber temperature sensitivity changes with the temperature [50]. Equation (2) can be further used to obtain a relation between the possible temperature measurement range under given conditions, such as the available optical spectrum. Let us assume that one has a measurement system that can operate in the range of $\lambda_{1}$ and $\lambda_{2}$ nm and has an FBG with $\lambda_{B}\left(T_{0}\right)=\lambda_{1}$, which was measured for a well-known temperature $T_{0}$. For such a configuration, the temperature measurement can be carried out in the range of $T_{0}$ to $T_{1}$, where $T_{1}$ can be calculated from Equation (3).

(3) $$T_{1}=\rho \left(\Delta \lambda_{B}\right),$$

where $\Delta \lambda_{B}=\lambda_{2}-\lambda_{1}$ and $\rho (\Delta \lambda_{B})$ is the inverse function of $\Delta \lambda_{B}\left(T\right)$, which can be calculated from Equation (4).

(4) $$\rho \left(\Delta \lambda_{B}\right)={\Delta \lambda_{B}}^{-1}\left(T\right)=\left\{\begin{array}{cc} \frac{\mp \sqrt{\alpha^{2}+4\beta \Delta \lambda_{B}}-\alpha }{2\beta }+T_{0} & \mathrm{if}\;\beta \neq 0\\ \frac{\Delta \lambda_{B}}{\alpha }+T_{0} & \mathrm{if}\;\beta =0\;\&\;\alpha \neq 0 \end{array}\right.$$.

**Figure 3:**
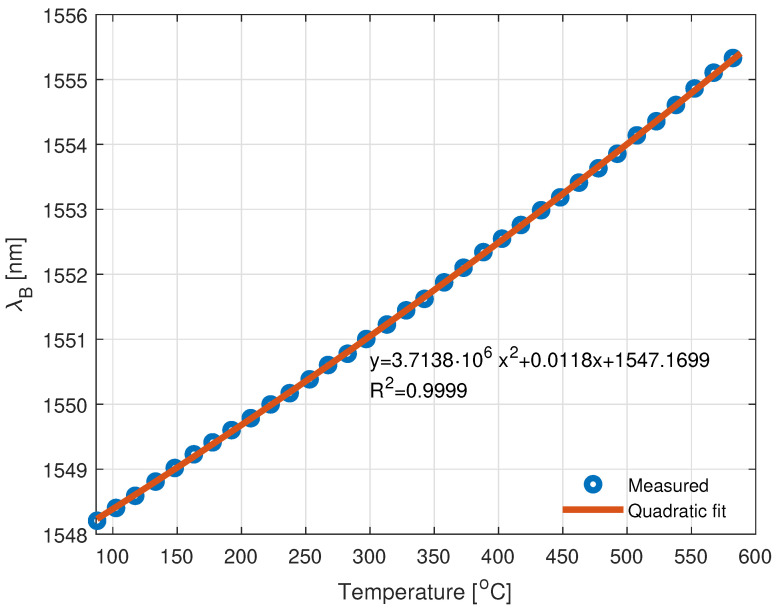
The dependency between FBG central wavelength and temperature induced by a hot-air soldering station. The $R^{2}$ of the quadratic fit is equal to 0.9999.

In order to obtain the α and β coefficients of the sensor, the dependence between the Bragg wavelength under different temperatures was measured and is presented in Figure 3. The measurement of a uniform femtosecond Bragg grating was carried out in a temperature range of 50 to 580 °C. Measurement was performed with the use of an InPhenix IPSDD1507 SLED as a BBS, an optical circulator, and a Yokogawa AQ6470D Optical Spectrum Analyzer (OSA). Fiber Bragg Grating was heated using the Weller WTHA 1 hot air station. The grating was glued with sodium silicate adhesive to the tip of the thermocouple, which was placed in the outlet of the hot air and served as a reference temperature measurement.

The utilized femtosecond FBG has an observable quadratic dependence between the Bragg wavelength shift and temperature, which proves the deliberations from [50,51,52]. The quadratic fit model obtained shows that the measured grating has $\alpha =0.0118\frac{\mathrm{nm}}{{}^{\circ }C}$ with $\beta =3.7138\cdot 10^{-6}\frac{\mathrm{nm}}{{}^{\circ }C^{2}}$ and these coefficients will be used in further analysis.

### 2.2. Optical Interrogator

**Figure 4:**
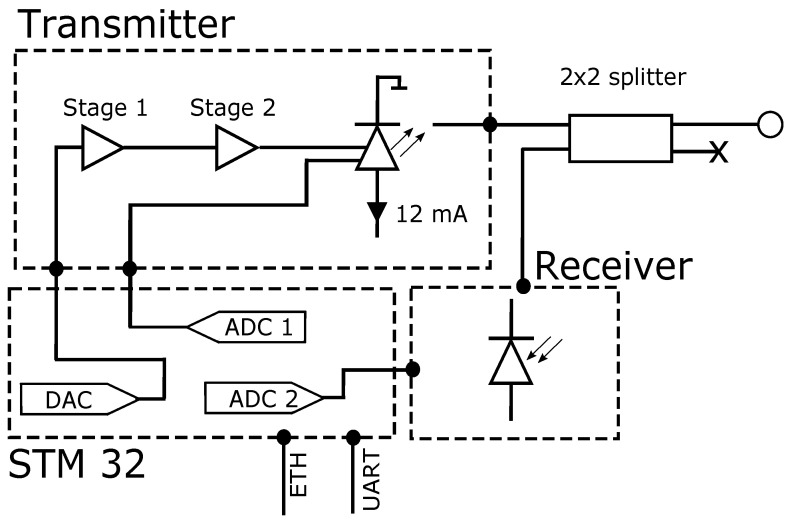
Schematic diagram of the developed interrogator. Two ADCs and one DAC of the microcontroller are utilized. The signal fed to the HCG input port is followed by a two-staged OPAMP. The signal from the photodetector is received by an ADC1, while ADC2 is utilized to measure the voltage on a thermistor built into the VCSEL. The laser is biased at 12 mA by a BJT-based circuit.

As indicated earlier, FBG-based sensors are naturally passive and thus must be stimulated by a light wave. The optical interrogator is responsible for illuminating the FBG, receiving the reflected signal, and then comparing the received and sent signals to evaluate the reflected spectrum. The proposed measurement device architecture is shown in Figure 4 and consists of a VCSEL, a photodetector, and a microcontroller.

**Figure 5:**
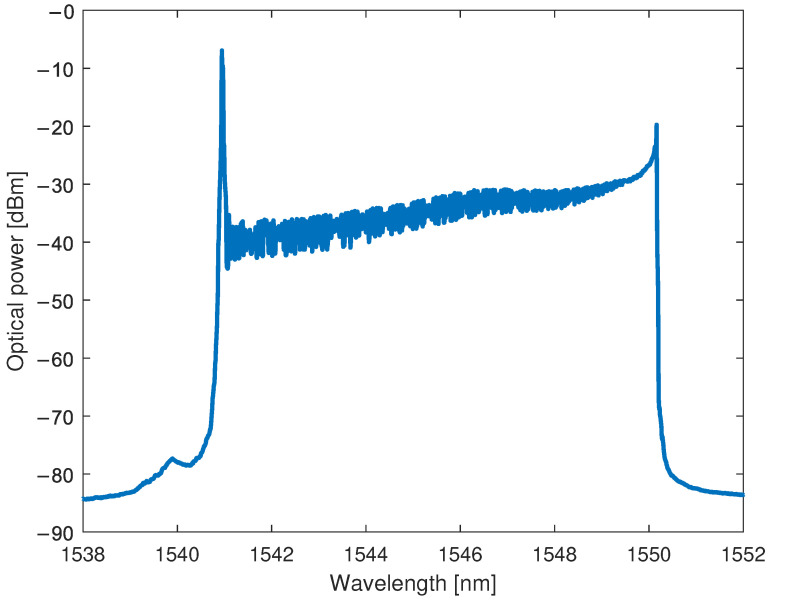
Spectrum of a continuously swept VCSEL-HCG laser. The laser was directly connected to the laboratory-grade OSA, and the measurement was performed with the utilization of long-time integration of the signal.

The VCSEL-HCG supplied by Bandwidth10 is working in the C-band and allows mode-hopping-free operation with constant output wavelength tuning achieved with HCG cavity [44] in the range of up to 10 nm and a speed up to hundreds of kHz, which makes it suitable for the interrogation of FBG sensors. The VCSEL bias is set with the stable current sink circuit based on the BJT transistor providing a current equal to 12 mA, which corresponds to the average output optical power of −3 dBm. Since the output wavelength of a VCSEL is changed with respect to the applied voltage to the HCG from a range of 0 V to −18 V, and the output of a DAC provides voltage levels from 0 to 3.3 V, a two-stage amplifier circuit was proposed that consists of two inverting OPAMPs. The first OPAMP is a buffer that is responsible for isolating DAC and VCSEL. The second stage amplifies DAC signal to the range of 0 to −18 V and provides a shift of the bias signal to a value of −9 V. The VCSEL has a Peltier module, but in order to decrease the power consumption of the device, it is not used. However, the built-in thermoresistor allows it to measure the operating temperature of the laser, which in turn is utilized to analyze the error in the calculation of the Bragg wavelength of the sensors. The spectrum of the laser wavelength swept in the full range is presented in Figure 5.

For the carried out sweeping speed equal to 427 Hz, one can obtain a measurement range from about 1540 to 1550 nm (edge to edge). The spectrum of a VCSEL laser may be misleading because of the relatively high power change between the lower and higher wavelengths in the operational range. Nevertheless, it can be explained by the fact that the tuning curve of a laser, i.e., the relation between the output wavelength and applied voltage to the HCG is quadratic [44]. However, the applied voltage is a signal with the shape of a linear ramp, and the spacing between the voltage values is constant. Thus, much more power is integrated by an OSA for the range of higher wavelengths.

In the proposed interrogator, a Koheron PD100 InGaAs photodiode was used. The transimpedance gain of the PD100 is equal to 3.9 kΩ, which allows for the receiving of signals with relatively low power while maintaining noise at an acceptable level.

The architecture is deliberately simplified in order to create as flexible a solution as possible. For example, if one wants to receive signals from distant sensors, it is possible to use a photodetector with a much higher transimpedance gain. Moreover, if a different wavelength range is required, one can simply change the VCSEL in such a way that it will suit specific needs.

The heart of the interrogator is an STM32 microcontroller, which is responsible for acquiring the operating laser temperature, setting the HCG voltage, and processing the signal received by the photodetector. Additionally, the microcontroller packages the signal together with the operational parameters of the components, such as the temperature of the VCSEL, in UDP/IP datagrams and sends them to the Control & Storage Unit. The data channel is physically isolated from the control channel to improve the reliability of the solution. The microcontroller communicates with the CSU via Ethernet to send measurement data. The UART-USB protocol is used to exchange the working parameters of the device.

In this work, it is assumed that STM32 is not involved in the advanced processing of the received signal. However, since STM32 has built-in math acceleration functionality, the capabilities of an interrogator can be easily extended with respect to the user’s needs. For example, if one wants to simplify the architecture of the whole measurement system, an STM32 will be more than perfect in order to perform offline Bragg wavelength computation.

As depicted in Figure 4, two built-in ADCs are utilized. The first—ADC1—gathers the signal from the photodetector, and the second—ADC2 is used for measuring the voltage on a VCSEL thermistor, which is then used to calculate the operating temperature of the laser. The control firmware is created in such a way that ADC1 and ADC2 are synchronized with DAC by the utilization of a built-in timer. DAC is used to feed the HCG with a ramp-shaped voltage signal. Timer interrupts are triggered with a 4 MHz clock. The utilized MCU has a 12-bit DAC, corresponding to 4096 possible voltage levels from 0 to 3.3 V. The positive slope of a ramp signal is assumed to use full DAC capabilities and consist of 496 samples, each of which corresponds to different voltage levels. Thus, the rise time of a ramp is equal to $\frac{4096}{4\;\mathrm{MHz}}=1.024\;\mathrm{ms}$. To maximize the interrogation speed, it is assumed that only one slope of a ramp is used, and thus the falling slope consists of only 512 samples. This value is an acceptable balance between maximum tuning frequency and not time-dawdling for undesirable operations, together with protecting the VCSEL from unwanted voltage spikes that can occur due to significant voltage changes over a short period of time.

To ensure that the photodetector receives the proper wavelength, one must analyze the maximal sensor distance. Since the timer signal is 4 MHz square, DAC can trigger on the rising edge and ADC on the falling edge of it. Thus, the formula for the maximal distance between the interrogator and the farthest sensor is $L_{MAX}=\frac{T_{set}\cdot c_{fiber}}{2}$, where $T_{set}$ is the time between setting the wavelength and starting acquisition reduced by the physical inertia of the HCG. In the presented system, the HCG setting time can be omitted because we only move by a small fraction of the wavelength in a range and the total tuning frequency is much lower than the maximum tuning frequency. The time between edges in the timer is equal to 125ns, so the maximal length is around 12.5 m, and the total length of patchcords used in the experiment fulfills this limit. Note that placing sensors at larger distances will result in errors in received power or even in a misleading wavelength.

In order to improve $L_{MAX}$, one can decrease the timer frequency, which will result in a longer time between setting certain wavelengths and measuring power, leading to an increase in the possible acquisition distance. Another option is to use parallelly connected sensors and ensure that each has a similar fiber length.

**Figure 6:**
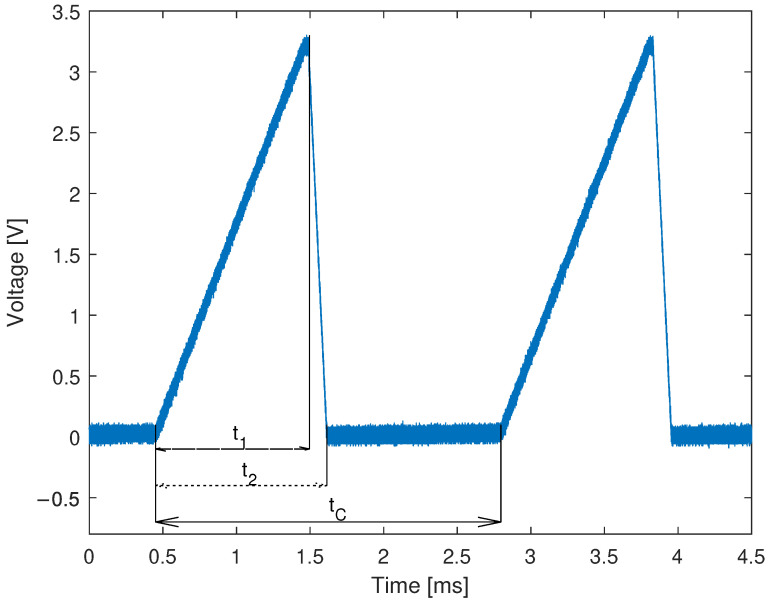
Voltage signal at the output of an STM32 DAC measured with LeCroy 202MXi oscilloscope. The ramp signal can be distinguished by three time-based parameters. The first $t_{1}$ is the rise time, the $t_{2}$ is the duration of a ramp, and the $t_{C}$ is the period of tuning signal.

The voltage ramp that is fed to the two-stage OPAMP circuit is shown in Figure 6.

From the measurements of an HCG driving signal, one can conclude that the positive sweeping time of a laser $t_{1}$ is equal to 1.024ms, the negative sweeping time $t_{2}-t_{1}$ is equal to 0.128ms, and finally the sweeping cycle $t_{C}$ is equal to 2.340ms, which transfers to an interrogating speed $1/t_{C}$ equal to 427Hz. The firmware collects data from the ADCs and packs them into UDP datagrams that are then sent to the CSU via an Ethernet connection. The time between the end of the first observable ramp and the second one is the time that is consumed by an MCU simply for creating encapsulation headers in a Lightweight TCP/IP (lwIP) stack and waiting until data are correctly sent to the CSU.

Based on the time analysis, it is possible to explain the high-power peak from the laser spectrum measurement shown in Figure 5 that occurs for approximately 1540 nm (corresponding to the 0 V on the HCG). The OSA integrates the optical signal in time and is not synchronized with the interrogator. Since the 0V state occurs more frequently than other voltages, much more power will reach OSA for wavelengths around 1540 nm. The presented interrogation scheme consists of a photodetector, which is used to collect optical power in time, and to overcome an integration-time problem, a rigorous synchronization between ADCs and DAC is implemented.

STM32 resources are used only for handling lwIP, because ADC and DAC data transfer is controlled by the Direct Memory Access (DMA) module. Accordingly, for more demanding applications, such as high-speed measurements, free resources can be used to calculate the Bragg wavelength, causing a significant drop in the size of the UDP payload, and then send them via IP and Ethernet. Such an approach could potentially decrease $t_{C}-t_{2}$ time and thus increase the interrogation speed.

### 2.3. Control & Storage Unit

Control & Storage Unit is a crucial element that is responsible for storing measurements and controlling the operation of an interrogator. The STM32 used in the interrogator can be capable of analyzing data from the sensors. However, when one has a capacious FBG network or wants to use FBG spectra for AI or ML classification, the potential of STM32 may be insufficient and thus, the utilization of CSU is necessary.

CSU software is built in the Python3 environment and is responsible for communicating with the interrogator and any external service or additional measurement system. The communication with an interrogator is utilized via both Ethernet and USB-UART protocols. When the measurement process is started, the Python app sends a configuration to the interrogator and makes sure that it is accepted by a measuring instrument. Subsequently, a communication channel via Ethernet with an interrogator is established. Afterward, the CSU app continuously gathers measurement data and assigns them appropriate timestamps.

### 2.4. Bypassing Limitations of Optical Interrogator

Based on the measurements carried out with an OSA presented in Figure 5, an effective operational spectral range of an interrogator is equal to circa 10 nm. Based on Equations (2) and (4) and parameters from Figure 3, for such a wavelength span, the temperature can be measured in the range of up to 760 °C. However, for some applications, such as power plant monitoring [53,54], or turbine engine examination [18,55,56], a much larger temperature range is required. To overcome such a limitation, one can either use two or more VCSELs in the interrogator unit or extend wavelength sweeping range through either a temperature or current manipulation. However, such solutions do not render the proposed system cost-effective or energy-efficient. Therefore, we propose another method to bypass the limitations of a VCSEL-HCG-based interrogator.

Let us assume that one wants to measure temperature from $T_{1}$ to $T_{2}$ and that the optical interrogator allows measuring wavelengths from $\lambda_{1}$ to $\lambda_{2}$. Furthermore, to avoid unwanted spikes in temperature calculation, the effective wavelength range is equal to $\gamma (\lambda_{2}-\lambda_{1})$. Note that the coefficient γ can theoretically be changed from 0 to 1. The lower γ, the more sensors are required to measure the desired temperature range. However, in the presented research, the value γ was chosen empirically and is assumed to be equal to 0.8, which gives a balance between the possible measurement range and the immunity to the appearance of unwanted spikes in the measurements and decreases the possible crosstalk between gratings [57]. Moreover, let us assume that the fiber sensors utilized change their wavelength with respect to Equation (2). From this, *K*—the number of gratings in the system can be calculated from Equation (5).

(5) $$K=\left\lceil \frac{\alpha \left(T_{2}-T_{1}\right)+\beta {\left(T_{2}-T_{1}\right)}^{2}}{\gamma (\lambda_{2}-\lambda_{1})}\right\rceil .$$

**Figure 7:**
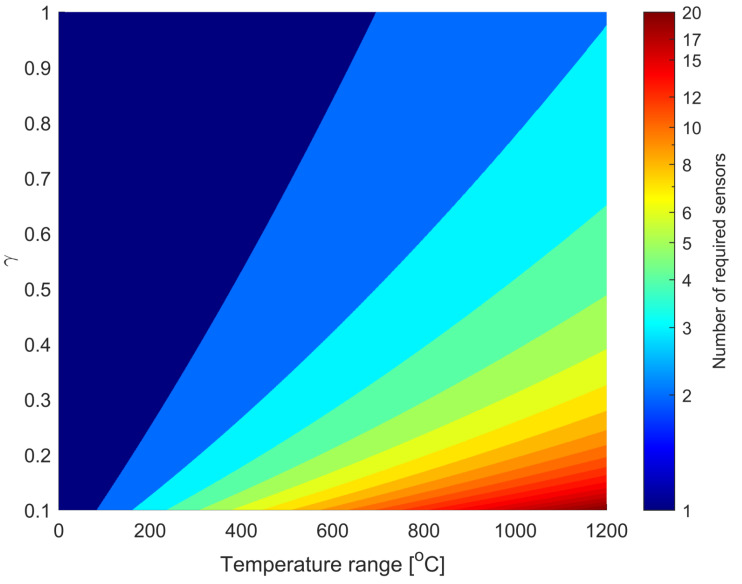
Relation between a number of gratings and the desired temperature measurement range.

The number of gratings required to measure the specific temperature range is presented in Figure 7 for $\lambda_{2}-\lambda_{1}=10$ nm, $\alpha =0.0118\frac{\mathrm{nm}}{{}^{\circ }C}$, $\beta =3.7138\cdot 10^{-6}\frac{\mathrm{nm}}{{}^{\circ }C^{2}}$, and γ=0.8.

**Figure 8:**
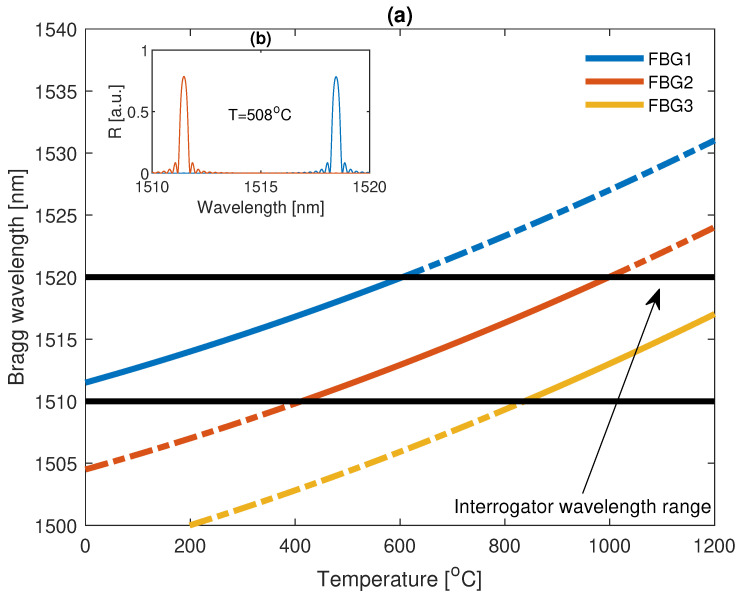
Concept of bypassing the limits of an optical interrogator. In (**a**), a relation between the Bragg wavelengths of the FBG sensors and the temperature acting on the fiber is shown. In (**b**), an example of a spectrum of two sensors for a temperature equal to 508 °C is shown.

Since the number of sensors required is well known, let us propose a method to bypass the limits of an optical interrogator, the concept of which is presented in Figure 8.

The concept is based on the assumption that each grating has the same properties when exposed to external factors, that is, each sensor has the same $\Delta \lambda_{B}\left(T\right)$. However, to maintain continuous temperature measurement, it is assumed that each *k*-th grating has a different central wavelength for the common reference temperature $T_{0}$. As depicted in Figure 8, since the coefficient γ is less than 1, for specific temperature ranges, the spectrum of two sensors can be observed in the interrogator range. This can improve the performance of the algorithm for Bragg wavelength calculation from the received signal, and prevent unwanted spikes in the measured temperature. The Bragg wavelength of a *k*-th grating can be calculated from Equation (6).

(6) $$\lambda_{B0}^{k}=\lambda_{1}+\left(\lambda_{2}-\lambda_{1}\right)\left(\frac{\left(1-\gamma \right)}{2}-\gamma \left(k-1\right)\right).$$

The temperature in the measuring environment can be calculated by taking into account Equation (6) and thus by analyzing the Bragg wavelength of the sensor. Recall that each sensor is chosen in such a way that it allows it to measure temperatures higher than the previous one. Therefore, one must bear in mind that the measured temperature considers the parameters of a specific *k*-th Bragg grating, which is suitable for measuring the current temperature in the environment. In order to properly apply the proposed method to the measurement system, the interrogator or CSU must remember the previous state of the system, especially the temperature at the earlier time. The temperature in the measuring environment can be calculated from Equation (7).

(7) $$T\left(t\right)=\rho \left(\lambda_{B}\left(t\right)-\lambda_{B0}^{k}\right),k:\left[\Delta \lambda \left(T\left(t-\delta t\right)\right),\Delta \lambda \left(T\left(t-\delta t\right)\right)+\gamma \left(\lambda_{2}-\lambda_{1}\right)\right]∋\left(\lambda_{B}\left(t\right)-\lambda_{B0}^{k}\right),$$

where $\lambda_{B}\left(t\right)$ is the measured Bragg wavelength of the sensor and T(t−δt) is the temperature obtained δt seconds ago. Note that when more than one sensor is observable in the interrogator wavelength range, one should take into account the $\lambda_{B}\left(t\right)$ that is closest to the previous one. It must be stated that the initial condition of the measurement, i.e., the value of T(0), must be well known.

## 3. Examination of the Concept

**Figure 9:**
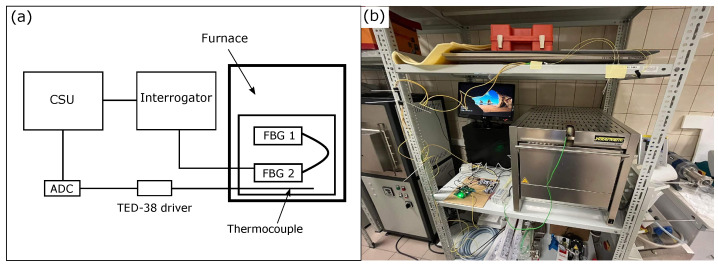
Architecture of the measurement bench (**a**), which consists of a CSU, an optical interrogator, two FBG-based sensors, and a thermocouple, which is considered to provide the reference temperature measurement. The measurement bench, together with the picture of the heating furnace, is presented in (**b**).

To examine the proposed concept, an exemplary measurement bench has been constructed, consisting of a Nabertherm N 11/HR heating furnace, an optical interrogator, CSU, two femtosecond Fiber Bragg grating sensors provided by FBGS, and a K-type thermocouple TP-202K-1b-400-1,0 with TED-38 driver, both supplied by Czaki. The measurement obtained from the commercial thermocouple is used as a reference to the measurement utilized with two FBGs in order to calculate a temperature error. The architecture of an exemplary system, together with the measurement bench, is shown in Figure 9.

The experiment was performed using femtosecond gratings, the parameters of which are shown in Table 1.

The concept is examined as follows. First, the FBGs together with the thermocouple are placed inside the furnace. It must be stated that, in order to minimize the influence of inhomogeneous temperature distribution inside the measuring chamber, sensors together with the thermocouple are placed as close as possible. Fiber Bragg gratings are serially connected to the interrogator, followed by an optical coupler. The STM32 NUCLEO-F746ZG and the built-in ADC are used to gather data from the TED-38 thermocouple driver, which is connected to the GPIO followed by a 1:3 voltage divider circuit. The CSU is responsible for synchronous data collection from the interrogator and thermocouple’s STM32. Synchronization between two devices is based on timestamps that are generated when the CSU receives data from either the interrogator or the thermocouple circuit.

The furnace parameters are such that the temperature is changed over 240 min from 25 to 1000 degrees with 100-degree steps. According to Equation (2), the temperature change in the furnace influences the change in Bragg wavelength, which must be properly determined.

### 3.1. Calculation of Central Wavelength

The measurement data from the interrogator contains both wavelength and received amplitude vectors, each of length *M*, denoted by X, and Y, respectively. One of the most common methods used to determine the central wavelength of the sensor spectrum is the centroid. The centroid method analytically finds the center of a mass of the analyzed spectrum. However, this method can be biased because of the noise present in the signal, and the relative spectral position of the sensor in the observable window. This is why the modified threshold centroid method is utilized, whose basis lies in the calculation of the center of the mass by taking into account only a specific set of measurement points. The selection is based on the reflected power of a grating and a relative threshold level denoted by η. Hence, the central wavelength of the analyzed grating can be calculated from Equation (8).

(8) $$\lambda_{C}=\frac{\sum_{i=1}^{M}Y\left[i\right]X\left[i\right]}{\sum_{i=1}^{M}Y\left[i\right]},i:Y\left[i\right]\geq \eta \max\left\{Y\right\}$$

**Figure 10:**
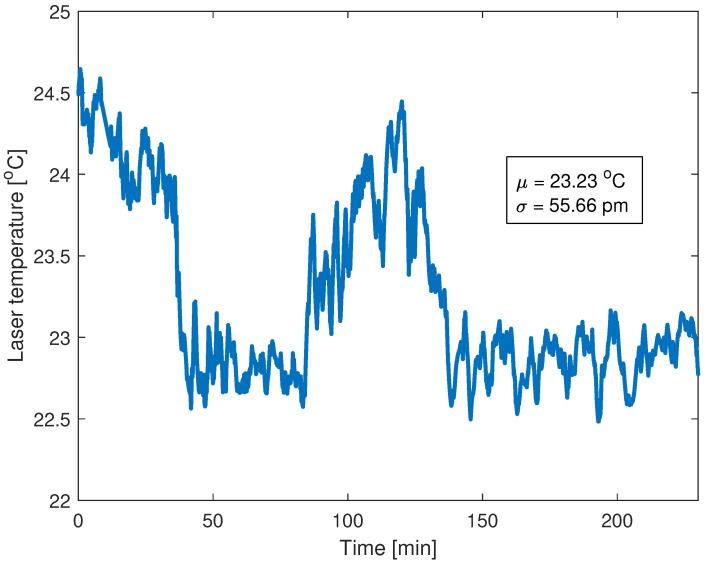
VCSEL-HCG laser temperature measured with a built-in thermistor. The standard deviation of a temperature change is equal to 0.5457 °C, which corresponds to 55.66 pm in output wavelength value [44].

In the experiment, the temperature of the laser is not stabilized, and its value over time is presented in Figure 10.

**Figure 11:**
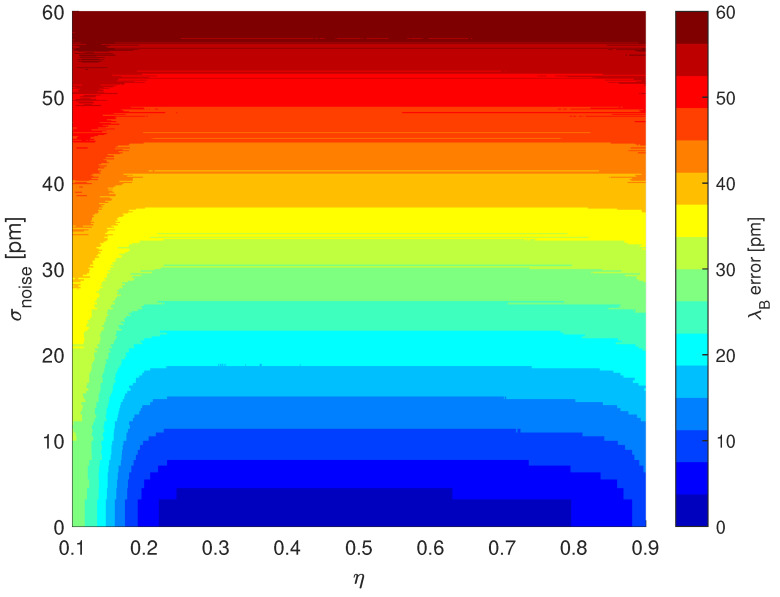
The relation between Bragg wavelength calculation error and laser wavelength instability ($\sigma^{2}$) together with the relative threshold level η utilized in the centroid method. The optimal value of η should be chosen in such a way as to minimize an error. Since the optimal point is related to the noise present in the system, one should properly analyze it before utilizing the centroid method. The simulations were performed in the MATLAB environment. The FBG spectrum was obtained using the TMM approach from [58]. The $\lambda_{B}error$ is calculated as the difference between the central wavelength obtained from Equation (8) and $\lambda_{max}$ from Equation (42) in [58].

The relatively high standard deviation of the laser’s temperature is caused by the fact that it is working in a highly unstable environment, which causes significant drops and increases in working temperatures and thus its output wavelength [44]. Since the Peltier module is not used, it is justified to analyze the centroid method by means of a wavelength noise. The relation between the Bragg wavelength calculation error and wavelength noise together with different η is shown in Figure 11.

One can conclude that apparently the higher the laser instability, the higher the Bragg wavelength error. Note that the selection of η value should be based on the analysis of grating reflectance and noise, whose main components are the noise of the photodetector and the eventual quantization error. However, a detailed analysis of a centroid method is beyond the scope of this article and has previously been demonstrated in detail [59,60,61].

**Figure 12:**
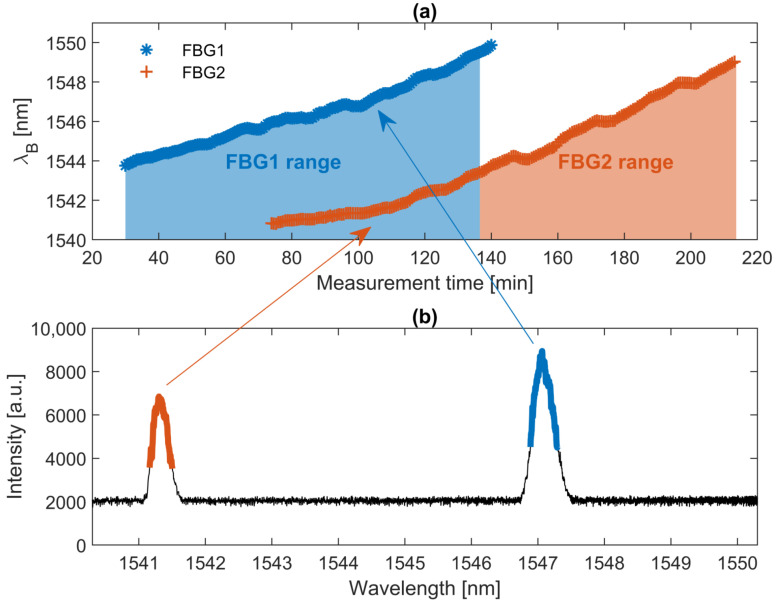
(**a**) Relation between the calculated central wavelength of the FBG sensors and the time of measurement. The asterisk curve represents the data from FBG1, and the plus sign line is for FBG2. (**b**) The spectrum obtained with the proposed interrogator for a temperature equal to circa $350{\;}^{\circ }C$ (the equivalent of the 105th minute of measurement).

On the basis of the analysis performed and the temperature variance obtained from the laser, a η=0.5 was assumed in the calculation of the central wavelength of the FBG. The calculated central wavelengths of two FBGs that were used to measure the temperature inside the furnace are presented in Figure 12.

### 3.2. Decreasing Error in the System

The analysis of a central wavelength calculation error demonstrated that undoubtedly such an error depends on the laser wavelength instability. Note that the temperature of the utilized laser was not controlled, and thus decreasing the influence of the wavelength instability and overall noise in the signal can improve an error of temperature retrieval. It can be demonstrated that the error can be minimized by taking the mean of the *N* measurements. Since the achieved interrogation speed is significantly greater than the changes of temperature in the furnace, a *N* can be relatively high and must satisfy the inequality (9).

(9) $$N\leq \frac{\delta_{\lambda }}{t_{C}\alpha_{T-\lambda }},$$

where $\delta_{\lambda }$ is the wavelength resolution of the interrogator, and $\alpha_{T-\lambda }=\max\left\{\alpha \frac{\partial T}{\partial t}+\beta {\left(\frac{\partial T}{\partial t}\right)}^{2}\right\}$.

To analyze analytically the variance of a mean signal over time, assume that the received signal y(t,λ) is the sum of the reflected power of the grating $R_{T}\left(t,\lambda \right)$ and Gaussian noise n(t,λ). Presently, let us assume that the signal y(t,λ) is averaged *N* times in time in such a way that the averaged signal is equal to $\frac{1}{N}\sum_{i=1}^{N}y\left(t_{i},\lambda \right))$, where $t_{i}$ is the *i*-th moment in time. Let us assume that in the period from $t_{1}$ to $t_{N}$, the signal $R_{T}\left(t,\lambda \right)$ is stationary and therefore has a variance equal to $\sigma_{R}^{2}$, and is not correlated with n(t,λ). The noise *n* is known to have a variance equal to $\sigma_{N}^{2}$. Based on this, one can achieve the formula for the variance of the averaged signal, as in Equation (10).

(10) $$Var\left(\frac{1}{N}\sum_{i=1}^{N}y\left(t_{i},\lambda \right)\right)=\sigma_{R}^{2}+\frac{1}{N}\sigma_{N}^{2}.$$

The gain in variance *G* after averaging is expressed as a relation between the unaveraged and averaged signals and can be calculated by Equation (11).

(11) $$G=\frac{\sigma_{R}^{2}+\sigma_{N}^{2}}{\sigma_{R}^{2}+\frac{1}{N}\sigma_{N}^{2}}.$$

### 3.3. Experiment Results

**Figure 13:**
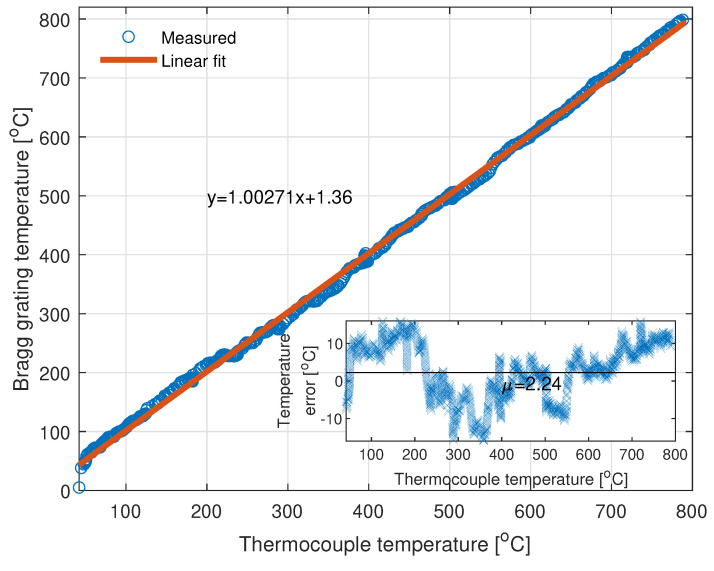
Temperature calculated with the FBG-based sensors versus temperature obtained from the thermocouple. The linear regression parameter $R^{2}=0.9988$.

On the basis of the concept presented in the previous sections, two FBGs from the measurement bench were utilized to jointly measure the temperature in the furnace. The relationship between the temperature obtained from the optical-based system and the thermocouple is presented in Figure 13.

The linear regression of the relationship in Figure 13 shows that there is a good correlation between the changes in temperature obtained from the gratings and measured by the thermocouple. However, the regression is biased by an error that originates from fluctuations in laser temperature and non-ideal compensation of a quadratic tuning curve of the laser. To overcome those issues, one can use a TEC circuit for the stabilization of a laser temperature and perform a more accurate characterization of the laser.

The temperature error is caused by the following factors: unstabilized temperature of the VCSEL, non-ideal compensation of the VCSEL laser tuning curve, positioning of the gratings in the heating furnace, and utilized peak calculation method. As stated above, the temperature of the laser was not stabilized in order to decrease the power consumption of the whole system. The peak-to-peak value of the laser temperature during the measurement process is equal to circa 1.96 ${}^{\circ }C$, which translates to the peak-to-peak value of the temperature error, measured by a potential FBG, equal to 17.84 °C. The peak-to-peak value obtained from the measurements is equal to 33.19 °C. Thus, the instability of the laser itself corresponds to about half of the final temperature error, and without proper temperature compensation, it is not possible to obtain a peak-to-peak value smaller than 17.84 °C. Note here that to compensate for the quadratic dependency of the tuning curve, its parameters must be known with the highest precision possible. The static curve is easy to obtain; however, the tuning characteristic changes with respect to the sweeping speed, and thus it is unambiguous to obtain proper quadratic fit parameters. In this research, static parameters were utilized to linearize the HCG-VCSEL response. That is why there is a non-zero error that originates from this fact in the final temperature measurement. In addition, there could be a slight misalignment of gratings and thermocouple which could have an effect to the final error. Finally, the weighted algorithm with thresholding was utilized for the calculation of the central wavelength of the sensor. Indeed, much more advanced method could potentially decrease the final error, but such an analysis lies beyond the scope of the article.

The proposed system allowed measuring the temperature inside the furnace from 25 °C to 800 °C. In the presented approach, the limitation of the system was induced by the fact that two off-the-rack gratings were used, so there was no possibility of tailoring the central wavelengths of the sensors to achieve a wider temperature range.

## 4. System Enhancement

In previous chapters, a measurement system was presented together with an experiment that demonstrated the possibility to measure the temperature up to 800 °C, which was limited by the utilized FBGs. According to the literature, femtosecond gratings inscribed in SiO_2_ glass can measure temperatures up to about 1100 °C [62]. However, with the utilization of specialized optical fiber Bragg gratings, such as sapphire-based (SFBG) ones, much higher temperatures can be measured, reaching up to 2000 degrees Celsius [63,64,65,66]. Such gratings are suitable for applications for which the possibility of measuring high temperatures is essential [19,53]. However, sapphire-based FBGs have a stronger Bragg wavelength and temperature dependence than standard fibers, and thus the value of $\alpha =0.023\frac{\mathrm{nm}}{{}^{\circ }C}$ and $\beta =4\cdot 10^{-6}\frac{\mathrm{nm}}{{}^{\circ }C^{2}}$ [63]. Hence, it is necessary to utilize more gratings in order to achieve the desired range of temperature measurement.

### Optimization of the System

For systems in which the increase in a Bragg wavelength as a function of temperature is large, it is necessary to use more FBGs, i.e., at least more than 3. Moreover, as concluded in the previous section, with increasing temperature, the relative measurement range of a specific grating for a constant interrogator wavelength range decreases, which is caused by a nonlinear response to the temperature. Thus, a continuous temperature estimate can be unambiguous. Furthermore, in the case of a blackout during measurement, the reconstruction of the state of the measurement bench can be nonexecutable. Henceforth, let us propose a reflectance-based multiplexation [67,68,69,70], which can allow one to determine an absolute temperature range of a sensor without knowing the previous state of the system. Namely, each utilized grating is assumed to have a unique reflectance and to be stable over time and non-dependant on the temperature.

Let us assume that the utilized photodetector is able to receive reflectances from 0 to 1. Next, assume that the reflectance values for each grating are linearly distributed. Then, a reflectance of a *k*-th grating can be calculated by Equation (12).

(12) $$R_{\max}^{k}=\frac{2k-1}{2K}.$$

**Figure 14:**
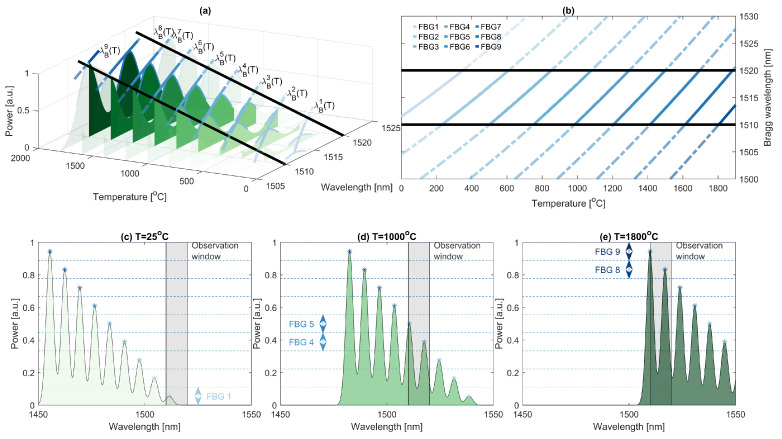
(**a**) Simulation of SFBGs spectra for different temperatures acting on sensors. The spectrum of each grating shifts with respect to the Equation (2). The black lines indicate spectral measurement range of the interrogator. (**b**) Bragg wavelength for each SFBG sensor in the function of a temperature. Spectrum received from all sensors for 25, 1000, and 1800 degrees Celsius is presented in (**c**), (**d**), and (**e**), respectively. The dashed lines in (**c**–**e**) represent reflectance confidence intervals.

In such an approach, the operational range, together with base parameters such as $T_{0}^{k}$ or $\lambda_{B0}^{k}$, can be deduced from a reflectance value by comparing the received power with the specific confidence intervals. For example, the reflectance of the first grating is in the range of 0 to 1/K, the second in the range from 1/K to 2/K, and so on. The principle of the system is similar to the concept presented in Section 3, but the grating number visible in the interrogator range depends on the received power and not the previous measurement. The concept is presented in Figure 14.

Each grating in the system is chosen in such a way that it is able to monitor a specific range of temperatures with respect to the interrogator wavelength range. The current temperature in the system can be calculated by Equation (13).

(13) $$T\left(t\right)=\rho_{k}\left(\lambda_{B}\left(t\right)-\lambda_{B0}^{k}\right),k:R\left(\lambda_{B}\left(t\right)\right)\in \left[\frac{k-1}{K},\frac{k}{K}\right],$$

where $\rho_{k}$ is the inverse function of the wavelength changes for the *k*-th grating with central wavelength equal to $\lambda_{B0}^{k}$, and $R(\lambda_{B}\left(t\right))$ is a current reflectance value. It should be noted that since sapphire optical fibers have a relatively large core area and air cladding, the obtainable FWHM of the gratings can reach up to a couple of nanometers [65], which can be problematic in a system with a small wavelength range that is close to the FWHM of the grating. However, it has been lately shown [71,72,73] that there is a possibility to obtain SFBGs having clearly lower FWHM than the spectral range in the proposed interrogator.

The most important remark that must be justified is the fact that state-of-the-art SFBG structures consist of up to 5 serially distributed gratings [74,75], which renders the proposed concept potentially inapplicable. Such limitation comes from the production process of SFBGs and the physical characteristics of the sapphire fiber, which causes a significant increase in the insertion loss of structures. However, there is a possibility to overcome such a limitation, where one can use parallelly connected sensors followed by an optical splitter.

As was previously reported, some types of gratings can change their reflectance or even central wavelength over time when exposed to high temperatures. However, to overcome such a problem, one can utilize the changing output power of the laser to compensate for the reflectance drop or modify its temperature to neutralize the changes of $\lambda_{B0}^{k}$, but such approaches will increase the power consumption of the system. Another possibility is the implementation of a digital correction that will compensate for the drop of the reflectance, through, for instance, monitoring of the exposure time of the gratings on high temperature.

## 5. Discussion and Conclusions

In this work, a cost-effective and fast interrogation system for fiber-based sensors was presented with a dedicated application in the field of high-temperature measurement. However, it is worth noting that the achieved operational wavelength range of the system is equal to approximately 10 nm. At first glance, such a feature renders the proposed system not competitive. However, the appropriate sensor network design and selection of initial central wavelengths of FBGs can lead to an increase in the system measurement range. Since the FBG production process is well established and has been mastered in a comprehensive way by many research groups, the need to adjust the sensors in the designed system is not an inconvenience [76].

An experiment with two femtosecond FBGs has been employed, which allowed for the examination of the concept. The results presented in the previous sections showed that with the utilization of different fiber Bragg gratings, the wavelength limitation of an interrogator is not a problem. Based on the approach of extending the measurement range of the system, a concept of multiplexation in both reflectance and wavelength domains was proposed for the most demanding applications.

The proposed optical interrogation system benefits from its simple architecture and possible flexibility in terms of adaptation to the desired needs, such as a specific temperature measurement range. The interrogator is based on continuous wavelength sweeping of a VCSEL-HCG laser. While in the experiment, a measurement speed was equal to 427 Hz, which resulted from the limitations introduced by the microcontroller. The tuning speed of the HCG-based VCSEL can reach hundreds of kHz and can be achieved with the utilization of, e.g., FPGA-based control of the system.

It is worth mentioning that the proposed concept for bypassing the limitations of the interrogator forces users to utilize more physical measuring points. Indeed, for precise spatial resolution, such an approach can be impractical. However, please note that the production cost of a single Bragg grating is, with a proper production site, negligibly small, and the number of gratings employed for sensing has an insignificant effect on the final costs of the system, which is essential for the industry. However, such a statement is valid only if one uses FBGs without housing. When the system designer intends to utilize proper housing, the sacrification of sensing points problem can influence the final price of the system. In such a situation, there is a wide field for optimization, especially around the concept that puts multiple FBGs in one housing. Surely, the presented approach forces system designers to optimize the possible positions of the sensors around the measurement point. Finally, if a higher spatial resolution is required, by means of higher measurement points, an optical switch can be employed to achieve a higher capacity of the sensing system, which allows to increase the number of sensors connected to the interrogation unit, as indicated in Figure 1.

The architecture of the interrogation system is suited for Industry 4.0 applications. Data are transferred through the Ethernet interface along with the utilization of the UDP and IP protocols. If one wants to achieve encrypted communication, an STM32 built-in cryptographic library can be used. The power consumption of an optical interrogator is equal to 3.5 W. However, the energy efficiency can be improved by decreasing the processor clock speed, or PCB design optimization along with the disposal of unnecessary components, such as diagnostic LEDs. What is more, the total costs of components that were used to build the interrogator can be optimized much below 2000 EUR, which is a considerable advantage over other commercial solutions available on the market.

The parameters of the proposed interrogating system are presented in Table 2. Since the tuning curve of the VCSEL, i.e., relation between output wavelength and applied voltage to the HCG structure is nonlinear, the calculation of the resolution parameter can be dubious. This is why it is calculated as the highest value of the derivative of the output wavelength versus the HCG voltage relation.

Since the measurement speed obtained may be insufficient for some applications, further work will focus on the utilization of an FPGA instead of an ARM-based controller to speed up the interrogator. In addition, future work will be associated with the implementation of a system for multipoint measurement of high temperatures in jet engines, which will help to detect the temperature in the combustion chamber. The proposed multiplexation scheme and the concept of bypassing the span limitation of the interrogator open the possibility of detecting a temperature gradient using many FBG sensors. It is worth noting that, in the future, a deep analysis of the noise present in the system must be performed to significantly reduce the temperature error obtained by the system. The measurement platform still needs a more reliable approach to calibrate both FBG sensors and the VCSEL laser.

## Figures and Tables

**Table 1:** Parameters of FBGs.

Grating	$\lambda_{B}$ @$T_{0}=25$ °C [nm]	R[%]
FBG 1	1544.8	70
FBG 2	1538.9	60

**Table 2:** Parameters of the proposed interrogator system.

Parameter Name	Value
Interrogation speed	427Hz
Wavelength range	10nm
Wavelength resolution	4.77pm
Communication	Ethernet & UART
Average power consumption	3.5W
Suppply voltage	9–18 V
Weight	≈200 g
Price	<2000 €

## Data Availability

Data underlying the results presented in this paper are not publicly available at this time but may be obtained from the authors upon reasonable request.

## Abbreviations

The following abbreviations are used in this manuscript:

ADC	Analog-to-digital converter
AI	Artificial intelligence
BBS	Broadband light source
CSU	Control & storage unit
DAC	Digital-to-analog converter
FBG	Fiber Bragg grating
GPIO	General-purpose input/output
HCG	High contrast grating
MCU	Microcontroller unit
ML	Machine learning
OPAMP	Operational amplifier
OSA	Optical spectrum analyzer
PD	Photodetector
SFBG	Sapphire fiber Bragg grating
SLED	Superluminescent diode
TEC	Thermoelectric cooler
TMM	Transfer matrix method
UART	Universal asynchronous receiver-transmitter
USB	Universal serial bus
VCSEL	Vertical cavity surface emitting laser
